# Assessments of Wind-Energy Potential in Selected Sites from Three Geopolitical Zones in Nigeria: Implications for Renewable/Sustainable Rural Electrification

**DOI:** 10.1155/2015/581679

**Published:** 2015-03-24

**Authors:** Joshua Olusegun Okeniyi, Olayinka Soledayo Ohunakin, Elizabeth Toyin Okeniyi

**Affiliations:** ^1^Department of Mechanical Engineering, Covenant University, Ota 112001, Nigeria; ^2^Department of Petroleum Engineering, Covenant University, Ota 112001, Nigeria

## Abstract

Electricity generation in rural communities is an acute problem militating against socioeconomic well-being of the populace in these communities in developing countries, including Nigeria. In this paper, assessments of wind-energy potential in selected sites from three major geopolitical zones of Nigeria were investigated. For this, daily wind-speed data from Katsina in northern, Warri in southwestern and Calabar in southeastern Nigeria were analysed using the Gumbel and the Weibull probability distributions for assessing wind-energy potential as a renewable/sustainable solution for the country's rural-electrification problems. Results showed that the wind-speed models identified Katsina with higher wind-speed class than both Warri and Calabar that were otherwise identified as low wind-speed sites. However, econometrics of electricity power simulation at different hub heights of low wind-speed turbine systems showed that the cost of electric-power generation in the three study sites was converging to affordable cost per kWh of electric energy from the wind resource at each site. These power simulations identified cost/kWh of electricity generation at Kaduna as €0.0507, at Warri as €0.0774, and at Calabar as €0.0819. These bare positive implications on renewable/sustainable rural electrification in the study sites even as requisite options for promoting utilization of this viable wind-resource energy in the remote communities in the environs of the study sites were suggested.

## 1. Introduction

At the year 2010, according to reports in [1], about 50% of the total population, globally, 60% of the population in Africa, 55% of the population in West Africa, and 79.441 out of the 158.423 million (i.e., 50.14%) of the people in Nigeria dwell in the rural areas. In the country, Nigeria, electricity generation capacity for the entire populace is most often lesser than 4000 MW in comparison to required energy demand that could be as high as 25,000 MW, and, due to this, 65% to 72% of the Nigerian rural populace lack access to electricity [2–4]. This lack of basic infrastructural requirement, on which major productive activities for developments in modern society are dependent [5, 6], leads to gross impoverishment of the populace in rural communities in Nigeria and in other developing countries with similar energy situations [2, 7, 8]. These affect socioeconomic well-being of the rural dwellers in the country [7, 9], a situation that is not much different from what is obtained in developing countries globally. For instance, estimations by the World Health Organization (WHO) indicated that annual premature death of women and young children attains 2.5 million while as high as 6% of global populace are infected with acute respiratory illness from hazardous gases and fumes emitted from traditional biomass stoves [9]. By the neglects of rural populace in Nigeria [5, 7–9], only 25% are economically active in agriculture, which is supposed to be their major occupation, 58% have no access to improved drinking water sources, and 72% have no access to improved sanitation facilities [1]. These are caused by energy-related problems. Many farmers are dissuaded from engaging beyond subsistence farming for lack of modern storage facilities which are energy dependent. Lack of electricity leads to lack of water pumping facility that could both ensure good drinking water and make water available for improved sanitation facility, which is, basically, a water-dependent hygienic issue.

It had been identified in studies [2, 4] that among the two options that could be employed for supplying electricity to the rural communities in developing countries, including Nigeria, the off-grid alternatives exhibited preference to the on-grid connection. The off-grid method of rural electrification becomes imperative due to the insufficiency of electricity supply available from the national grid and the remoteness of the rural communities from the grid which leads to prohibitive cost of electric-power installations, maintenance, and operations [2, 4, 7]. For the off-grid electrification option for the rural areas in the country, adoption of renewable energy portends potential of suitability for the remote communities that could be sustainable with the additional advantage of reducing unwholesome environmental effects from the use of fossil fuel sources.

**Figure 1:**
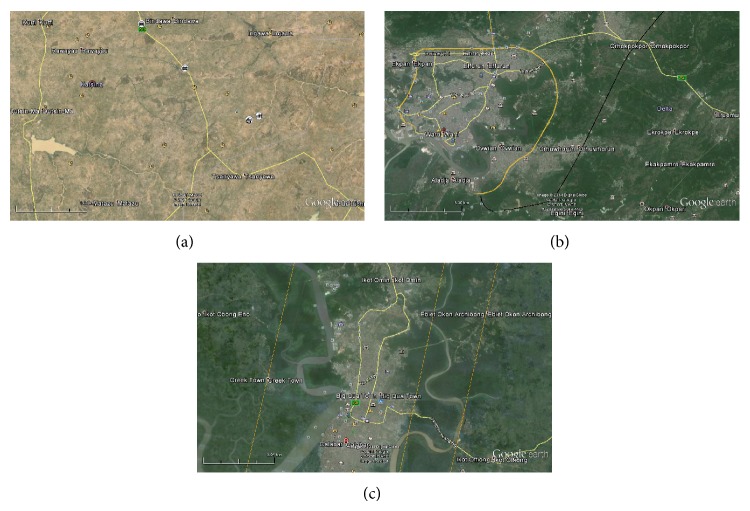
The study sites and environs in Nigeria: (a) Katsina, Katsina State; (b) Warri, Delta State; (c) Calabar, Cross River State.

A viable renewable energy option that could find applicability for most regions of remote rural communities in the country includes the use of wind energy for generating electricity for the electrification of the rural areas. However, useful exploitation of energy from wind sources for a region requires assessment of the wind-energy potential in the environs of such region for requisite knowledge of energy availability as well as viability of installing wind-energy systems in such locations for electric-energy generation. While many studies [5, 10–17] have deliberated on wind characteristics and energy potential from different parts of the country, none have undertaken such study for establishing how the wind energy could have been applicable as a solution for rural-electrification problems in the country. Therefore, the objective of this study was to investigate wind-energy potential of selected sites from three geopolitical zones in Nigeria for the purpose of gaining insights into the usage of wind-energy systems for sustainable rural electrification in the selected parts of Nigeria. For this, selected sites employed include Katsina from Katsina State, in the northern part, Warri from Delta State, in the southwestern part, and Calabar from Cross River State, in the southeastern part of the country. Aerial images showing topologies of these study sites and environs are shown in Figure 1. Motivation for these study sites was from the consideration that environs of these locations are characteristically identified with rural communities characterised with nonavailability of electric power due to their nonconnection to the national grid of electricity supply in the country [3, 7, 18]. Also, Katsina represents a high altitude location compared to Warri which is a low altitude site while the altitude of Calabar lies between that of Katsina and Warri. These make the study sites of interests for investigating how the different modes of wind-energy potential in the sites could be assessed for solving rural-electrification problems in the environs of the study sites and in other similar regions of the world.

## 2. Materials and Methods

### 2.1. Description of Selected Sites and Wind-Speed Data

Daily wind-speed data that were measured from January 2006 to December 2010 were obtained from Nigerian Meteorological Agency (NIMET), Abuja, Nigeria, for the selected sites of Katsina, from northern, Warri from southwestern, and Calabar from the southeastern parts of Nigeria. In these locations, the meteorological stations were, respectively, located as given in Table 1, which also includes the air density, *ρ* (kg/m^3^), at each of the stations. The wind-speed data were captured in each of the stations using cup-generator anemometer at a height of 10 m.

### 2.2. Statistical Distribution Analyses of Wind-Speed Data

For studying the statistics for describing wind-speed data, the measured wind speed was subjected to the extreme-value distribution fittings of the Gumbel and of the Weibull probability density functions (pdfs). This followed recommendations for suitability study of probability density function for describing wind-speed data by [19] but, in spite of this, there is paucity of studies in which the Gumbel pdf has been employed for detailing wind-speed frequency.

The Gumbel distribution [21, 22] employed for fitting variable of wind-speed data *x* in this study has its cumulative distribution function given by the following expression:

(1) $$\begin{array}{c} F\left(x\right)=1-\exp\left\{-\exp\left[-\left(\frac{x-\lambda }{\beta }\right)\right]\right\}, \end{array}$$

where *λ* ≡ the location and *β* ≡ the scale parameters that were estimated, from *n* sample sized wind-speed data, from regression of the linearized cumulative distribution function which assumes the following form:

(2) $$\begin{array}{c} \ln\left\{-\ln\left[1-F\left(x\right)\right]\right\}=-\frac{x}{\beta }+\frac{\lambda }{\beta }. \end{array}$$

Estimated *λ* and *β* values from this were employed for evaluating Gumbel mean, *μ*_*G*_, of the wind speed from the following expression [21–23]:

(3) $$\begin{array}{c} v_{m,G}\equiv \mu_{G}=\lambda -\beta \Gamma^{\prime }\left(1\right), \end{array}$$

where Γ′(1) = *γ*, which is Euler's constant and the value of *d*Γ(*n*)/*dn* when this differential is evaluated at *n* = 1.

In similar manner, Weibull distribution [5, 6, 21, 24–26] utilized for fitting variable of wind-speed data *x* in this study has its cumulative distribution function given by the following expression:

(4) $$\begin{array}{c} F\left(x\right)=1-\exp\left\{-{\left(\frac{x}{\beta }\right)}^{\eta }\right\}, \end{array}$$

where *η* is the shape and *β* is the scale parameters [21, 23, 27–29] that were evaluated, from *n* sample sized wind-speed data, from regression of the linearized cumulative distribution function in the following form:

(5) $$\begin{array}{c} \ln\left\{-\ln\left[1-F\left(x\right)\right]\right\}=\eta \ln x-\eta \ln\beta . \end{array}$$

Estimated values of *η* and *β* obtained from this were also used for evaluating the Weibull mean, *μ*_*W*_, of the wind speed from the following expression [10, 14, 29–32]:

(6) $$\begin{array}{c} v_{m,W}\equiv \mu_{W}=\beta \Gamma \left(1+\frac{1}{\eta }\right), \end{array}$$

where Γ(·) is the gamma function of (·).

### 2.3. Fitting Performance of Probability Distribution Models

Criteria employed for evaluating fitting performance of the probability distribution models include the correlation coefficient, *R*, and the Nash-Sutcliffe coefficient of efficiency, CoE [15, 33, 34]. These are, respectively, estimated from the formula

(7) $$\begin{array}{c} R=\frac{\sum_{i=1}^{n}\left(o_{i}-\overset{-}{o}\right)\left(p_{i}-\overset{-}{p}\right)}{\sqrt{\sum_{i=1}^{n}{\left(o_{i}-\overset{-}{o}\right)}^{2}}\times \sqrt{\sum_{i=1}^{N}{\left(p_{i}-\overset{-}{p}\right)}^{2}}},\\ \text{CoE}=1-\frac{\sum_{i=1}^{n}{\left(o_{i}-p_{i}\right)}^{2}}{\sqrt{\sum_{i=1}^{n}{\left(o_{i}-\overset{-}{o}\right)}^{2}}}, \end{array}$$

where *o* and *p* are the observed and the predicted values of the wind-speed data.

### 2.4. Wind-Power Modeling and Electric-Power Simulation

The mean wind-power density (W/m^2^) available at the anemometer height of 10 m could be calculated for each pdf as [5, 31]

(8) $$\begin{array}{c} P_{m}=\frac{1}{2}\rho v_{m,\text{pdf}}^{3}. \end{array}$$

Also, electric-power output, *P*_*e*_, simulation employs the rated electric power *P*_*eR*_ for wind turbine model through the following expression [5, 10, 13]:

(9) $$\begin{array}{c} P_{e}=\left\{\begin{array}{cc} 0 & v<v_{C}\\ P_{eR}\left(\frac{v^{\eta }-v_{C}^{\eta }}{v_{R}^{\eta }-v_{C}^{\eta }}\right) & v_{C}\leq v\leq v_{R}\\ P_{eR} & v_{R}\leq v\leq v_{F}\\ 0 & v>v_{F}, \end{array}\right. \end{array}$$

where *v*_*C*_, *v*_*R*_, and *v*_*F*_ are the cut-in, the rated, and the cut-off speeds, respectively, of the wind turbine model. Evaluation of the average power output *P*_*e*,ave_ of the wind turbine model, for determining total energy production and the total income from the wind-energy conversion system, was obtained from [5]

(10) $$\begin{array}{c} P_{e,\text{ave}}=P_{eR}\left(\frac{e^{-{\left(v_{C}/\beta \right)}^{\eta }}-e^{-{\left(v_{R}/\beta \right)}^{\eta }}}{{\left(v_{R}/\beta \right)}^{\eta }-{\left(v_{C}/\beta \right)}^{\eta }}-e^{-{\left(v_{F}/\beta \right)}^{\eta }}\right). \end{array}$$

It is worth noting that the simulation of electric-power output *P*_*e*_ in 9 and average power output *P*_*e*,ave_ in 10 was for wind speed measured at height *h*_0_ = 10 m. For simulating these quantities for different hub heights *h* of wind turbine model, the extrapolations of scale and shape parameters of the pdfs, *β*_0_ and *η*_0_ at the measurement height *h*_0_ = 10 m, to the hub height *h* are required. This could be obtained from [5, 35]

(11) $$\begin{array}{c} \beta \left(h\right)=\beta_{0}{\left(\frac{h}{10}\right)}^{\varepsilon },\\ \eta \left(h\right)=\frac{\eta_{0}}{\left[1-0.088\ln\left(h/10\right)\right]}, \end{array}$$

where the exponent *ε* was evaluated from

(12) $$\begin{array}{c} \varepsilon =\frac{\left[0.37-0.088\ln\beta_{0}\right]}{\left[1-0.088\ln\left(h/10\right)\right]}. \end{array}$$

From the simulated wind-energy system, the capacity factor evaluation employs the ratio of the averaged turbine power to the turbine rated power [5, 24, 36]:

(13) $$\begin{array}{c} C_{f}=\frac{P_{e,\text{ave}}}{P_{eR}}. \end{array}$$

### 2.5. Analyses of Econometric Implications and Prospects of Wind-Energy System

The analyses of the econometric implication and prospect of wind-energy system development in the study sites employ computation of the present value cost, PVC, for the price *y* of a given turbine over the lifetime *t* of the turbine operation from the following equation [5, 10, 35, 37, 38]:

(14) PVC=y1+rC+ytrOMR1+irI−i×1−1+i1+rIt −rSC·y1+rC×1+i1+rIt,

where *r*_*C*_ = rate of the price of turbine for civil/structural works and other connections, *r*_OMR_ = annual rate of the price of turbine for operation, maintenance, and repair, *i* = interest rate, *r*_*I*_ = inflation rate, and *r*_SC_ = rate of the price of turbine and civil work for the scrap value of the turbine after the expiration of the turbine lifetime. By these, the cost per kWh of electricity generation through the wind turbine system was obtained from [5]

(15) $$\begin{array}{c} {\text{Cost}}_{\left(\text{per}\;\text{kWh}\right)}=\frac{\text{PVC}}{P_{e,\text{ave}}\times t}. \end{array}$$

## 3. Results and Discussion

### 3.1. Estimated Parameters for the Study Sites

**Figure 2:**
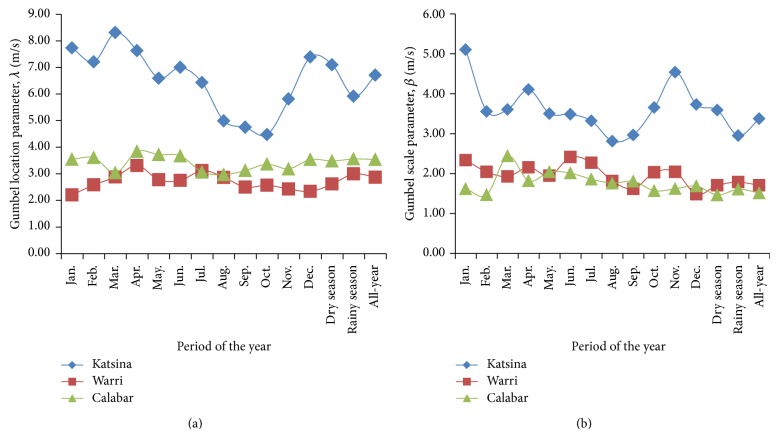
Estimated parameters of the Gumbel distribution model for the study sites: (a) Gumbel location parameter and (b) Gumbel scale parameter.

**Figure 3:**
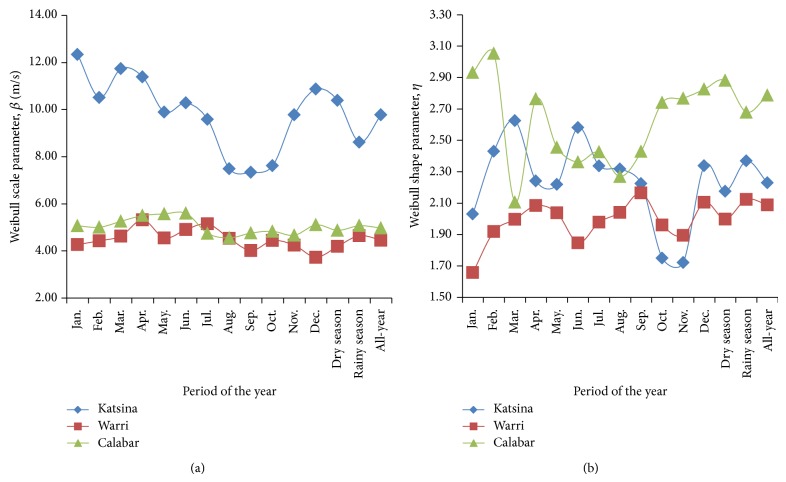
Estimated parameters of the Weibull distribution model for the study sites: (a) Weibull scale parameter and (b) Weibull shape parameter.

Plots of the estimated parameters of the Gumbel and the Weibull distribution functions are presented in Figures 2 and 3, respectively, for the monthly, seasonal, and all-year (January–December) considerations, all from 2006 to 2010. For this, it is worth noting that rainy season in Katsina, northern Nigeria, spans through June to September, four months, while the remaining eight months constitute dry season in the prevalent Sahel (tropical dry) climate. In contrast, rainy season spans through March to July and then September to October while dry season spans through November to March and a short dry season in August, termed “August break,” in the tropical rain forest (or equatorial monsoon) to which the southern part of Nigeria, thus Warri and Calabar, belongs. From Figure 2, it could be observed that the location parameters of the Gumbel pdf fittings of Katsina wind-speed data were of higher value than the location parameters obtained from the fittings from Warri and from Calabar in all the periods of the years studied. Specifically, the Gumbel location parameter, Figure 2(a), ranged from 4.48 m/s in October to 8.31 m/s in March at Katsina but from 2.20 m/s in January to 3.31 m/s in April at Warri or from 2.97 m/s in August to 3.61 m/s in April at Calabar. Also, the Gumbel scale parameter, Figure 2(b), ranged from 2.81 m/s in August to 5.10 m/s in January at Katsina, while it ranged from 1.62 m/s in September to 2.42 m/s in June at Warri or from 1.41 m/s in February to 2.44 m/s in March at Calabar. By these results, the Gumbel parameters bare suggestions of similarities of wind-speed characteristics in the study sites of Warri in the southwestern and of Calabar in the southeastern Nigeria.

Figure 3 also showed that the scale parameters of the Weibull pdf fittings of Katsina wind-speed data were of higher value than those from the wind-speed data fittings from Warri and from Calabar. The Weibull scale parameter, Figure 3(a), ranged from 7.34 m/s in September to 12.34 m/s in January at Katsina while it ranged from 3.73 m/s in December to 5.32 in April at Warri or 4.66 m/s in November to 5.60 m/s in June at Calabar. However, unlike the statistical parameters considered thus far, the Weibull shape parameters, Figure 3(b), estimated from the wind-speed data from Calabar overshot those estimated from the wind-speed data in Warri, for all periods of the year, and from Katsina, for 9 out of the 12 months, the dry, rainy, and the all-year wind speed. The Weibull scale parameter ranged from 2.11 in March to 3.05 in February at Calabar while it ranged from 1.72 in November to 2.63 in March at Katsina or from 1.66 in January to 2.17 in September at Warri.

These Weibull shape parameter values are of specific statistical importance because they bare indications of the uniformity of the wind-speed data in the study sites. Thus, the higher Weibull shape parameters at Calabar indicated higher slope of the Weibull fitting model for this study site in 5 and also implied that the modeled wind speed at Calabar exhibited more uniformity than the modeled wind speed from the other sites.

### 3.2. Performance Model of the Distribution Fittings of Wind-Speed Data

**Figure 4:**
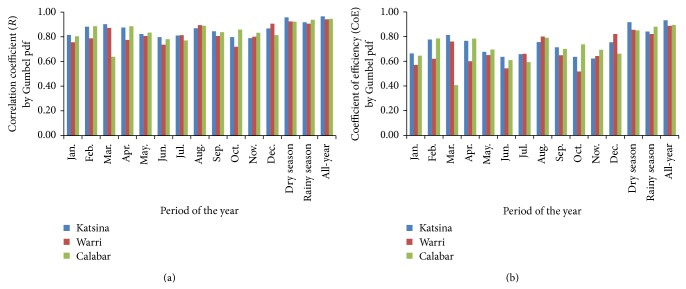
Performance modeling of the Gumbel distribution fitting of wind-speed data for the study sites. (a) Correlation coefficient (*R*) and (b) Nash-Sutcliffe coefficient of efficiency (CoE).

Plots of the performance model of the fittings of wind-speed data in the three study sites by the Gumbel distribution are presented in Figure 4, using evaluated values of correlation coefficient, *R*, shown in Figure 4(a), and of Nash-Sutcliffe coefficient of efficiency, CoE, shown in Figure 4(b). This showed that, by the monthly consideration, correlation coefficient and coefficient of efficiency (*R*, CoE) of the Gumbel pdf fitting of Katsina wind-speed data ranged from 78.90%, 62.25% in November to 90.23%, 81.42% in March. These bare indications that the modeling efficiency of the wind-speed data in Katsina by the Gumbel pdf model ranged from “good” efficiency model in November to “excellent” efficiency model in March, as per the model efficiency classification from [39]. By that classification in [39], the Gumbel fitting of the rainy season wind-speed data in Katsina at *R*, CoE of 91.75%, 84.19% also indicated “very good” efficiency model. However, the modeling efficiencies of the Gumbel pdf fittings of the dry season and the all-year wind-speed data in Katsina indicated “excellent” efficiency model at the *R*, CoE of 95.74%, 91.66% for the dry season and 96.57%, 93.27% for the all-year period.

The Gumbel fitting of wind-speed data in Warri ranged from *R*, CoE of 71.95%, 51.77%, indicating “good” model efficiency, in October to 90.65%, 82.18%, indicating “very good” model efficiency, in December. Also, the dry season, rainy season, and the all-year wind speed in Warri were fitted by the Gumbel pdf at the respective *R*, CoE of 92.48%, 85.53%; 90.61%, 82.11%; and 94.17%, 88.67%, all of which are classified to the “very good” model efficiency. The Gumbel fitting of Calabar wind-speed data ranged from *R*, CoE of 63.81%, 40.72% in March to 88.89%, 79.01% in August. By these, the fitting model of March wind speed in Calabar is classified to “fair” model efficiency while that of August is classified to “very good” model efficiency. In similar manner, the dry season, rainy season, and all-year wind speed were fitted at “very good” model efficiency by the Gumbel pdf *R*, CoE of 92.18%, 84.97%; 93.86%, 88.09%; and 94.60%, 89.50%, respectively.

**Figure 5:**
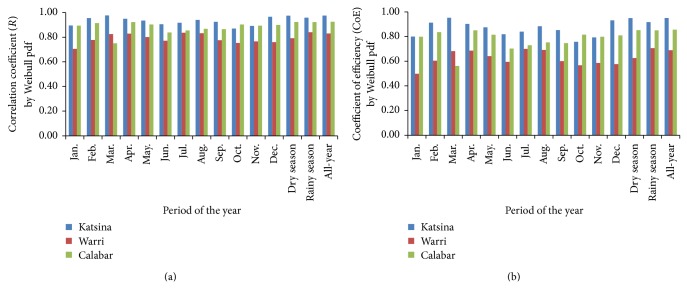
Performance modeling of the Weibull distribution fitting of wind-speed data for the study sites. (a) Correlation coefficient (*R*) and (b) Nash-Sutcliffe coefficient of efficiency (CoE).

Performance models of the Weibull fitting of wind-speed data for the three study sites are presented in Figure 5, where estimations of correlation coefficient, *R*, are plotted in Figure 5(a) and estimations of Nash-Sutcliffe coefficient of efficiency, CoE, are plotted in Figure 5(b). These showed that performance model of Weibull fitting of monthly wind-speed data ranged from (87.07%, 75.81%) “very good” in October to (97.62%, 95.31%) “excellent” in March at Katsina but from (70.56%, 49.78%) “fair” in January to (83.67%, 70.00%) “good” in July at Warri.

The Weibull fitting performance model of monthly wind-speed data ranged from (74.98%, 56.21%) “good” in March to (92.27%, 85.13%) “very good” in April at Calabar. By similar considerations, performance models of the Weibull fitting of seasonal and all-year wind-speed data are classified as “excellent” at Katsina, “good” at Warri, and “very good” at Calabar.

### 3.3. Mean Wind-Speed and Wind-Power Density Models for the Study Sites

**Figure 6:**
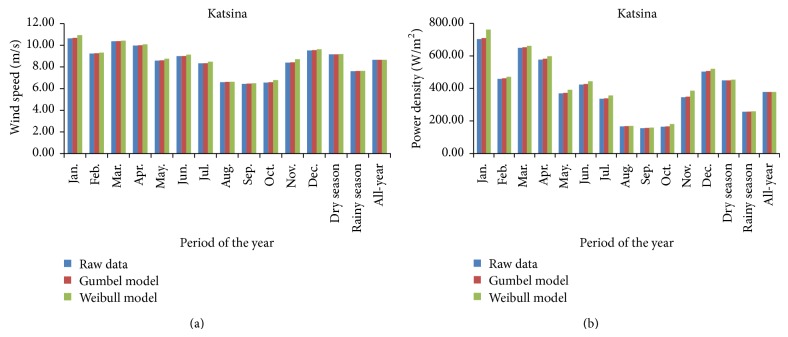
Wind-speed and power density models for Katsina: (a) mean wind-speed plots and (b) mean power density plots.

The mean wind-speed and mean-power density models for Katsina are presented in Figure 6 for the monthly, seasonal, and all-year analyses of wind-speed data by the probability distribution models.

From the figure, it could be observed that Katsina, in northern Nigeria, was windier in the months constituting its dry seasons, with January being the windiest month, than in the months of its rainy season where September was the least windy month. Thus, monthly mean wind speed and mean power density, in the form (raw data, Gumbel model, and Weibull model), ranged from (6.44, 6.46, and 6.50) m/s and (155.60, 157.12, and 159.80) W/m^2^ in September to (10.65, 10.68, and 10.94) m/s and (703.15, 709.93, and 761.92) W/m^2^ in January. The mean wind speed and power density for the windier dry season were (9.17, 9.17, and 9.20) m/s and (449.12, 449.73, and 454.10) W/m^2^; for the rainy season were (7.61, 7.62, and 7.64) m/s and (257.11, 257.75, and 259.55) W/m^2^; and for the all-year model were (8.66, 8.66, and 8.66) m/s and (377.76, 378.12, and 378.46) W/m^2^.

**Figure 7:**
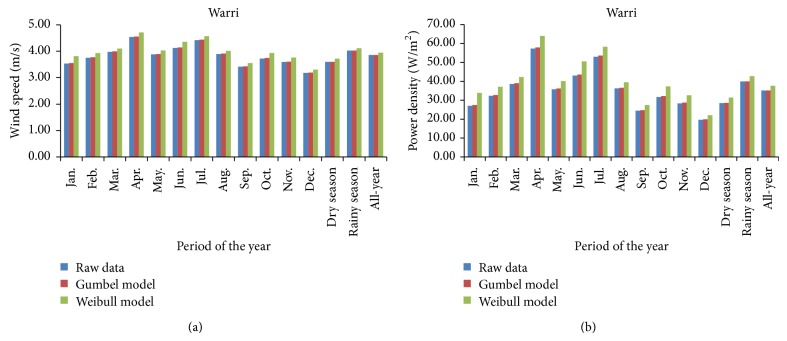
Wind-speed and power density models for Warri: (a) mean wind-speed plots and (b) mean power density plots.

The probability distribution models of the mean wind-speed and mean power-density models for Warri are presented in Figure 7 for the monthly, seasonal, and all-year analyzed wind-speed data.

From the figure, it could be deduced that the wind-speed model in Warri, in southwestern Nigeria, patterned differently, especially in the seasonal consideration, from what is obtained in the Katsina model. Unlike the wind-speed model in Katsina, the rainy season was windier than the dry season in Warri and the windiest month was April, a month in the rainy season at Warri, while the least windy month was December, a month in the dry season at Warri. At Warri, monthly mean wind speed and power density also in the form (raw data, Gumbel model, and Weibull model) ranged from (3.18, 3.19, and 3.30) m/s and (19.68, 19.90, and 22.05) W/m^2^ in December to (4.54, 4.56, and 4.71) m/s and (57.32, 57.88, and 64.00) W/m^2^ in April. The mean wind speed and power density for the windier rainy season were (4.02, 4.03, and 4.12) m/s and (39.91, 39.98, and 42.70) W/m^2^; for the dry season were (3.60, 3.60, and 3.72) m/s and (28.49, 28.57, and 31.41) W/m^2^; and for the all-year season were (3.86, 3.86, and 3.95) m/s and (35.13, 35.18, and 37.64) W/m^2^.

**Figure 8:**
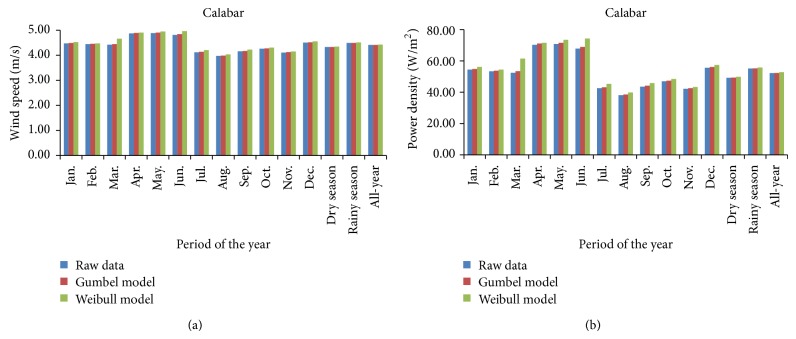
Wind-speed and power density models for Calabar: (a) mean wind-speed plots and (b) mean power density plots.

Mean wind-speed and mean power-density models for Calabar are presented in Figure 8 also for the monthly, seasonal, and all-year modeling of wind-speed data by the statistical distribution fitting functions. The figure also showed that the rainy season in Calabar, in southeastern Nigeria, was windier than the dry season, thus finding similarity with what was obtained at Warri in southwestern Nigeria. This bares supports for the prepotency of windiness of the rainy season over the dry season in the tropical rain forest (equatorial monsoon) climate predominant both in Warri and in Calabar, Nigeria. However, while the probability distribution fittings had exhibited agreements thus far, in Katsina and in Warri, at identifying the windiest month, the windiest month by the raw data and Gumbel pdf reckonings differs from what is identified by the Weibull pdf. While the raw data and the Gumbel pdf models identified May, with mean wind speed of 4.88 m/s (raw data) or 4.90 m/s (Gumbel), as the windiest month, the month of June was identified as the windiest month by the Weibull pdf at its modeled mean wind speed of 4.96 m/s (Weibull). These months of May and June, which both fall within the rainy season at Calabar, have mean power densities of 70.80 W/m^2^ (May, by raw data model) or 73.44 W/m^2^ (May by the Gumbel pdf model) and 74.28 W/m^2^ (June, by the Weibull pdf model). In spite of these, all the statistical models identified August, a dry season month in Calabar, the “August break,” as the least windy month with wind speed and power density of 3.97 m/s and 38.06 W/m^2^ (raw data) or 3.99 m/s and 38.55 W/m^2^ (Gumbel) or 4.03 m/s and 39.87 W/m^2^ (Weibull). The mean wind speed and power density for the windier rainy season were (4.49, 4.49, and 4.51) m/s and (55.09, 55.21, and 55.75) W/m^2^; for the dry season were (4.32, 4.33, and 4.34) m/s and (49.19, 49.29, and 49.82) W/m^2^; and for the all-year season were (4.41, 4.41, and 4.42) m/s and (52.21, 52.27, and 52.72) W/m^2^, in the form (raw data, Gumbel model, and Weibull model).

## 4. Simulations and Econometric Implications of Electric-Power Generation

### 4.1. Wind Turbine System and Electric-Power Output Simulations

The foregoing results of modeled wind speed and power density identified Katsina with wind-speed class that could be favourable for electricity generation from wind turbine system application while Warri and Calabar were identifiable as low wind-speed sites [5, 40]. However, this study is not deliberating on the comparison of the study sites but on investigating how the available wind in each of the sites could find usefulness for electricity generation in the remote areas. By this it is worth noting that usage of conventional wind turbine could be highly cost intensive for off-grid connections desired for the remote rural communities in the study sites, including Katsina, in spite of the higher wind modeled for that northern geopolitical region. Based on these considerations, a low-cost wind turbine system with the added advantage of low cut-in wind speed, such that it would be also applicable to the low wind-speed sites, was idealized for electricity generation from the wind-power in the three study sites. The Honeywell BTPS6500 wind turbine by WindTronics [5] having the characteristics presented in Table 2 finds suitability for this criterion.

In spite of the identification of this low cut-in wind turbine system to the study sites, it is worth noting from Table 2 that even the mean wind speed of the higher-class wind modeled for Katsina still falls short of the 13.9 m/s rated wind speed of the idealized wind turbine system. That this rated wind speed would be required for the delivery of the rated power of the applied wind turbine system necessitates investigating further hub heights, *h*, for improving the wind speeds at the study sites towards the turbine rated wind speed and for improving wind-power output. This was done by requisite applications of 9 to 12 and values in Table 2 to the Weibull modeled results and by these applications, the power simulation from heights *h* = 10 m to 110 m is presented in Table 3. It should be noted that, for this important wind-power simulation, the Gumbel model could not be used because of its intrinsic lack of shape parameter, *η*, which is a required quantity for electric-power simulation from wind in 9 and 10.

**Figure 9:**
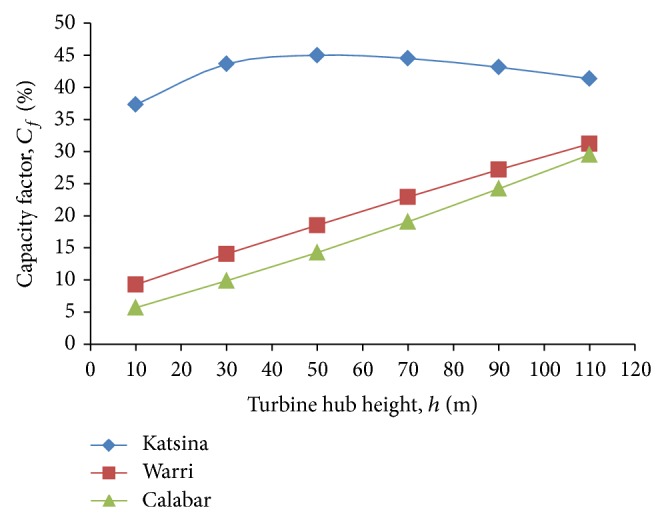
Capacity factor against turbine hub height for the all-year simulation in the study sites.

The power output simulations from the wind turbine system in Table 3 showed that the electric power *P*_*e*_ that could be generated increased with turbine hub heights, *h*, for each of the periods studied and in each of the study sites. At Katsina, in northern Nigeria, the electric-power output even exhibited potency of surpassing the rated power of the turbine system at the turbine hub height *h* = 110 m, at which *P*_*e*_ = 1714 W compared to the turbine *P*_*eR*_ = 1500 W. In spite of these, the average power output, *P*_*e*,ave_, that kept increasing with increasing hub heights at Warri and at Calabar in all the periods studied, peaked at Katsina when hub height *h* = 50 m and kept decreasing thereafter, in the years 2006 to 2008 and the all-year model. Averaged power output, *P*_*e*,ave_, also peaked at Katsina in the year 2009 when hub height *h* = 70 m and decreased thereafter, thus, leaving only the year 2010 as the only year in which the average power output kept increasing with hub heights of the wind turbine system at Katsina. These patterns of simulated power output are further highlighted by the plots of capacity factor, *C*_*f*_, against turbine hub height, *h*, for the all-year model of each of the study sites presented in Figure 9. Apart from the power output patterns, it could also be deduced from the figure that the capacity factors of the low wind-speed sites, Warri and Calabar, tend towards those of the higher wind-speed class, Katsina, as the turbine hub height increases.

### 4.2. Econometric Implications of Electric-Power Generation

For the econometric modeling applications to the study sites, using 14, the price, *y*, for the selected BTPS6500 wind turbine and its smart box inverter/controller system was set at *y* = €4, 200.02 ≡ ₦932, 469.67 and the turbine lifetime was set at *t* = 20 years [35]. Other assumed parameters necessary for the requisite applications of 14 for econometric analyses were presented in Table 4. This includes the special indication that a progressive increase of 5% was utilized for *r*_*C*_, the rate of turbine price for civil/structural works and other connections, at each hub height increment for catering for additional materials that may be required for increasing the wind turbine height, *h*.

Econometrics implications of the modeled wind-energy potential, obtained from substituting values from Tables 2 and 4 into 10 and 14 with other modeled results as required, are presented in Table 5 for turbine hub heights ranging from 10 m to 110 m, at each of the study sites. These tabulated econometric implications further support the results from electric-power simulations from the modeled wind speed by the continuous increase of the present value cost, PVC, for all the study sites, with increasing hub height of the wind turbine system. For Warri and Calabar, annual power output and the total power output, through the turbine service life, *t* = 20 years, were also increasing with increasing hub heights of the wind turbine system. In contrast to these, the annual power output and the total power output increased as the turbine height increased from 10 m to 50 m at which the output power simulations peaked and started to decrease thereafter as the turbine hub height further increases. These bare implications of the 50 m as the optimal hub height of the wind turbine model for optimum electric-power generation at Katsina.

**Figure 10:**
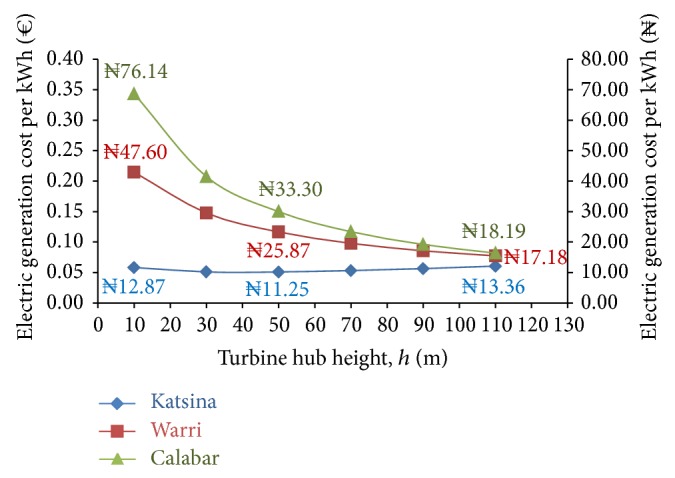
Costs of electricity generation per kWh in each of the study sites.

From the foregoing tabulated values of present value cost and total power output, the modeled cost of generating 1 kWh of electrical power at the various turbine heights was evaluated using 15 and the results from these evaluations are presented in Figure 10.

This figure showed that the cost of generating 1 kWh of electricity, at Katsina, decreased with increasing hub height from €0.0580 ≡ ₦12.87 when *h* = 10 m until *h* = 50 m where it was €0.0507 ≡ ₦11.25. Further increase in turbine hub height from this 50 m culminated in increased cost/kWh of electricity generation which attained €0.0602 ≡ ₦13.36 as the hub height increased to *h* = 110 m. This further supports the considerations from the electric-power output simulations that the turbine hub height *h* = 50 m was the optimum hub height for favorable generation of electricity power, also potent with the least cost/kWh electricity, from the wind resource at Katsina, northern Nigeria.

In furtherance of this, Figure 10 also showed that the cost/kWh of electric-power generation from Warri and from Calabar, which were indicated by the modeled wind speed and mean power density as low wind-speed sites, decreased with increasing hub height of the wind turbine system. At Warri, cost/kWh model for electricity generation continuously decreased, with increasing turbine hub heights, from €0.2144 ≡ ₦47.60 at *h* = 10 m to €0.0774 ≡ ₦17.18 at *h* = 110 m. At Calabar, cost/kWh modeled for generating electric power also decreased continuously with increasing turbine heights from €0.3429 ≡ ₦76.14 at *h* = 10 m to €0.0819 ≡ ₦18.19 at *h* = 110 m. From these, it could be noted that large discrepancies in electric-power generation costs ensuing from well-pronounced differences in wind-speed class models still culminated in converging cost/kWh model of electric-power generation with requisite increase in turbine hub heights.

## 5. Implications of the Modeled Wind-Energy Potential for Renewable Rural Electrification

The modeled costs of electric-power generation, ₦11.25/kWh at Katsina, ₦17.18/kWh at Warri, and ₦18.19/kWh at Calabar, bare potency of cheap/affordable electricity generation from wind that could be used as off-grid solution for the electrification of the rural communities at the study sites. This form of electricity generation from renewable wind-resource energy will not only constitute a sustainable form of clean/green electric power for the remote rural areas predominant in the environs of the study sites but will also bare potencies of added advantages, some of which include

- amelioration of energy poverty in the populace of the rural communities by the readily available and affordable electric power which could be generated at the affordable cost/kWh of electricity modeled for the study sites;
- improvement of the socioeconomic well-being of the populace where the electric generation could be utilized for improving access to potable water and access to water for sanitation purposes, for such needed water resource could now be pumped from boreholes;
- improved commercial activities in the rural communities where the available electricity power could be employed for agricultural produce storage/preservation through electric refrigeration techniques as well as utilization of the electricity for other small scale commercial activities prevalent in the rural communities, for example, tailoring/fashion designs, hairdressing and saloon, and catering/refreshments as well as relaxation centers;
- improvements in the living standards, conditions, and comforts of the dwellers in the rural communities, through such access to usage of electric fans, electric washing machines, electric lightings at nights, and radio and television powered from clean renewable/affordable energy, which could stem rural-urban migration and reduce pressures on facilities even in the urban centers;
- potency of enabling environments for attracting industries that could in turn improve wealth and well-being in the rural communities through, for instance, job availability and other corporate social responsibilities that could attend locating such industries in the community such as improved access roads and transportation systems and establishment of schools and health centers for their members of staff that would eventually serve the general community.

Although the modeled wind-resource energy from this study constitutes affordable clean and sustainable electric power from renewable source, the initial cost could still be cost intensive for the current rural populace in the environs of the study sites. However, many options could be suggested for surmounting this initial cost and ensuring rural electrification using the wind-energy resource in these rural communities. Some of the options that could be suggested include

- joint cooperative actions by the rural dwellers that could take the form of contributions or launching for attracting donations for the procurement/installation of the wind turbine system for the clean and renewable wind-resource energy being proposed in this study;
- formation or initiation of public-private partnership with governments or the grassroots arm of government available in the environs of the rural communities whereby both the community and the government jointly fund the procurement/installation of the proposed wind-resource energy system;
- partnership with other corporate financing bodies/institutions that could produce the initial funding for the procurement/installation of the wind turbine system and with whom payback of the fund could be designed, say with mild interest; such payback could take the form, for example, of electric bills payment say at the double of the cost/kWh modeled for the electricity generation from wind-energy, for this would also be affordable, by the dwellers of the rural community to such bodies/institutions; this payback could be sustained until the pay-off of the initial fund and the requisite mild interest after which the renewable energy generating system could totally belong to the community;
- provision of land properties by the rural communities for industries, for example, agroprocessing and other allied industries, in exchange for corporate social responsibilities by such industries that would include provision of the proposed wind-resource turbine system for renewable electric energy generation. These industries themselves could use such clean/affordable electric power for running their operations as well as for the electrification of the rural communities in which the industries are sited.

## 6. Conclusions

Potentials of wind-energy resources from three geopolitical zones in Nigeria, Katsina in Northern Nigeria, Warri in southwestern Nigeria, and Calabar in southeastern Nigeria, were assessed in this paper for investigating how the wind energy could be used for solving rural-electrification problem. Results showed that the wind speed, by raw data and by Gumbel and Weibull models, respectively, ranged from 6.44, 6.46, and 6.50 m/s to 10.65, 10.68, and 10.94 m/s at Katsina; 3.18, 3.19, and 3.30 m/s to 4.54, 4.56, and 4.71 m/s at Warri; and 3.97, 3.99, and 4.03 m/s to 4.88, 4.90, and 4.96 m/s at Calabar. These wind speed models and estimated power densities from the models identified Katsina as a high wind-speed class, while Warri and Calabar were identified by the models as low wind-speed sites. However, econometric analyses using wind turbine system at various hub heights showed that the cost per kWh of electricity at the three study sites tends to be converging at increasing hub height of the wind turbine system. These eventually culminated in cheap/affordable and sustainable models of electricity power generation from the clean and renewable wind resource in the environs of the study sites by which cost/kWh of electricity generation at Kaduna = €0.0507, at Warri = €0.0774, and at Calabar = €0.0819. Advantages that could accrue from such renewable energy generation from wind and the suggestions for surmounting the initial cost-intensive funding for procurement/installation of the wind turbine system were detailed in the study.

## Figures and Tables

**Table 1:** Description of meteorological stations at the selected sites.

S/No.	Site	Latitude (°N)	Longitude (°E)	Altitude (above sea level) (m)	Air density, *ρ*, (kg/m^3^)
1	Katsina	13.01	07.41	517.6	1.1653
2	Warri	05.31	05.44	6.1	1.2243
3	Calabar	04.58	08.21	61.9	1.2179

**Table 2:** Characteristics of the wind turbine model BTPS6500 system.

Characteristics	Hub height, *h*, (m)	Blade diameter (m)	*v*_*c*_ (m/s)	*v*_*R*_ (m/s)	*v*_*F*_ (m/s)	*P*_*eR*_ (W)
Value	10	1.7	0.2	13.9	17.0	1500

**Table 3:** Annual and all-year simulations of electric-power output at various turbine hub heights in the study sites.

Location	Period	*h* = 10 m		*h* = 30 m		*h* = 50 m		*h* = 70 m		*h* = 90 m		*h* = 110 m	
		*P*_*e*_ (W)	*P*_*e*,ave_ (W)	*P*_*e*_ (W)	*P*_*e*,ave_ (W)	*P*_*e*_ (W)	*P*_*e*,ave_ (W)	*P*_*e*_ (W)	*P*_*e*,ave_ (W)	*P*_*e*_ (W)	*P*_*e*,ave_ (W)	*P*_*e*_ (W)	*P*_*e*,ave_ (W)
Katsina	2006	594.15	595.16	892.36	668.21	1160.14	671.11	1426.30	651.17	1698.98	622.03	1982.01	589.36
	2007	770.20	631.02	1132.89	652.48	1450.87	629.56	1761.74	596.39	2075.95	560.97	2398.30	526.11
	2008	451.36	542.15	740.06	687.38	1017.79	722.66	1307.77	713.84	1617.06	684.67	1949.45	646.34
	2009	434.31	510.50	662.90	639.88	872.75	684.04	1084.56	691.52	1304.28	679.83	1534.82	657.70
	2010	358.94	429.31	515.11	555.42	654.10	619.67	791.08	652.84	930.40	666.70	1074.09	667.82
	All-year	522.29	559.58	778.56	654.69	1008.66	675.01	1237.29	667.53	1471.43	647.05	1714.38	620.28

Warri	2006	72.84	98.82	118.44	164.35	164.63	230.60	214.57	300.90	269.40	374.27	329.85	447.98
	2007	114.93	151.77	176.57	238.51	236.08	319.68	298.19	398.27	364.41	472.06	435.56	538.36
	2008	119.20	149.09	169.11	217.26	215.11	278.74	261.43	337.98	309.34	394.84	359.47	448.29
	2009	137.64	171.08	193.47	246.69	244.41	313.05	295.32	374.93	347.67	432.23	402.16	484.14
	2010	127.99	161.31	183.15	236.80	234.12	304.36	285.56	368.42	338.86	428.54	394.73	483.54
	All-year	107.96	139.16	159.66	211.08	208.60	277.97	258.89	343.80	311.81	407.78	368.02	468.16

Calabar	2006	65.52	90.15	110.08	154.79	156.27	221.78	207.09	294.33	263.69	371.35	326.89	449.82
	2007	81.59	110.95	133.15	185.13	185.31	259.65	241.66	337.51	303.50	416.66	371.65	493.44
	2008	66.64	91.81	112.24	158.00	159.57	226.65	211.66	300.88	269.73	379.42	334.60	458.98
	2009	64.29	88.62	108.47	152.77	154.41	219.48	205.06	291.96	261.59	369.15	324.80	448.04
	2010	54.81	76.19	94.61	134.32	136.78	196.04	183.92	264.54	237.15	339.43	297.27	418.30
	All-year	61.72	85.33	104.94	148.21	150.15	214.04	200.22	286.01	256.30	363.19	319.22	442.65

**Table 4:** Parameters employed for econometric analyses.

Parameter	*r*_C_^*^	*r*_OMR_	*i* ^†^	*r*_*I*_^†^	*r*_SC_
Value (%)	20	25	6.25	11.8	10

^*^Increased progressively by 5% at each hub height increment.

^†^Sourced from [20].

**Table 5:** Econometrics implications of electric-power generation from modeled wind at study sites.

Hub height *h* (m)	Katsina			Warri			Calabar		
	PVC (€)	Annual power output *P*_*e*,ave_/yr (kWh)	Total power output *P*_*e*,ave_ × *t* (kWh)	PVC (*€*)	Annual power output *P*_*e*,ave_/yr (kWh)	Total power output *P*_*e*,ave_ × *t* (kWh)	PVC (€)	Annual power output *P*_*e*,ave_/yr (kWh)	Total power output *P*_*e*,ave_ × *t* (kWh)
10	5,500.02	4,744.68	94,893.60	5,500.02	1,282.58	25,651.52	5,500.02	801.91	16,038.21
30	5,702.44	5,612.30	112,246.01	5,702.44	1,933.54	38,670.87	5,702.44	1,375.33	27,506.69
50	5,904.86	5,828.98	116,579.70	5,904.86	2,534.15	50,682.99	5,904.86	1,968.56	39,371.29
70	6,107.27	5,791.70	115,833.97	6,107.27	3,119.44	62,388.83	6,107.27	2,609.11	52,182.23
90	6,309.69	5,631.27	112,625.44	6,309.69	3,682.58	73,651.69	6,309.69	3,286.77	65,735.31
110	6,512.10	5,408.99	108,179.77	6,512.10	4,208.84	84,176.73	6,512.10	3,974.56	79,491.15
